# Role of Inflammatory Mediators, Macrophages, and Neutrophils in Glioma Maintenance and Progression: Mechanistic Understanding and Potential Therapeutic Applications

**DOI:** 10.3390/cancers13164226

**Published:** 2021-08-23

**Authors:** Abdul Samad Basheer, Faridah Abas, Iekhsan Othman, Rakesh Naidu

**Affiliations:** 1 Jeffrey Cheah School of Medicine and Health Sciences, Monash University Malaysia, Jalan Lagoon Selatan, Bandar Sunway 47500, Malaysia; abdul.basheer@monash.edu (A.S.B.); iekhsan.othman@monash.edu (I.O.); 2 Laboratory of Natural Products, Faculty of Science, University Putra Malaysia (UPM), Serdang 43400, Malaysia; faridah_abas@upm.edu.my; 3 Department of Food Science, Faculty of Food Science and Technology, University Putra Malaysia (UPM), Serdang 434000, Malaysia

**Keywords:** inflammatory mediators, neuroinflammation, tumor-associated macrophages, tumor-associated neutrophils, signaling pathways, glioma

## Abstract

**Simple Summary:**

The tumor microenvironment is a complex network comprised of neoplastic and a variety of immune cells, proteins, and inflammatory mediators. Previous studies have shown that during cancer progression, diverse inflammatory molecules, either directly or indirectly via recruiting immune cells, support the process of carcinogenesis. The present review focuses on the mechanistic understanding of the oncogenic role of these inflammatory mediators and immune cells, particularly tumor-associated macrophages (TAMs) and tumor-associated neutrophils (TANs) in glioma maintenance and progression. Moreover, the potential therapeutic benefits of targeting inflammatory mediators, immune cells, and associated signaling pathways in glioma genesis have also been discussed.

**Abstract:**

Gliomas are the most common, highly malignant, and deadliest forms of brain tumors. These intra-cranial solid tumors are comprised of both cancerous and non-cancerous cells, which contribute to tumor development, progression, and resistance to the therapeutic regimen. A variety of soluble inflammatory mediators (e.g., cytokines, chemokines, and chemotactic factors) are secreted by these cells, which help in creating an inflammatory microenvironment and contribute to the various stages of cancer development, maintenance, and progression. The major tumor infiltrating immune cells of the tumor microenvironment include TAMs and TANs, which are either recruited peripherally or present as brain-resident macrophages (microglia) and support stroma for cancer cell expansion and invasion. These cells are highly plastic in nature and can be polarized into different phenotypes depending upon different types of stimuli. During neuroinflammation, glioma cells interact with TAMs and TANs, facilitating tumor cell proliferation, survival, and migration. Targeting inflammatory mediators along with the reprogramming of TAMs and TANs could be of great importance in glioma treatment and may delay disease progression. In addition, an inhibition of the key signaling pathways such as NF-κB, JAK/STAT, MAPK, PI3K/Akt/mTOR, and TLRs, which are activated during neuroinflammation and have an oncogenic role in glioblastoma (GBM), can exert more pronounced anti-glioma effects.

## 1. Introduction

Glioma, which is one of the deadliest malignancies, develops within the central nervous system (CNS) and affects primarily the adult population. These neoplasms are broadly classified as diffuse gliomas, which have the capacity to infiltrate into CNS parenchyma. In contrast, the other group of gliomas represented by pilocytic astrocytomas and ependymal tumors have a restricted growth pattern [1]. Worldwide, every year, about ~100,000 people are diagnosed with the diffuse gliomas [2,3]. Among the gliomas, glioblastoma (GBM), a Grade IV category of tumor, is the most lethal and accounts for 70–77% of primary brain malignancies [4,5], which will be discussed in detail in this review.

Recently, an association between chronic inflammation and carcinogenesis has been demonstrated in several experimental models and clinical conditions [6,7]. Chronic inflammatory response resulting from persistent injury or infections can eventually lead to cancer (e.g., *Helicobacter pylori* infection may lead to gastric cancer and mucosal lymphoma). Other studies have reported triggering of inflammatory responses by cancerous cells, oncogenes (e.g., Ras and Myc), and tumor-associated macrophages, particularly in the microenvironment [8]. The inflammation-enriched tumor microenvironment (TME) has been shown to be responsible for the progression of developing tumors into highly malignant neoplasms, including GBM [9].

Neuroinflammation-enriched TME is created due to the production of pro-inflammatory cytokines, chemokines, and growth factors. The role of inflammatory mediators is also crucial in creating an immunosuppressed microenvironment, thus increasing the survival capacity of glioma cells. In gliomas, these players are responsible for enhanced proliferation, invasion, and/or maintenance of GBM cells stemness. Various inflammatory mediators secreted from glioma cells directly contribute to cancer cell expansion and invasion and assist in the recruitment of non-neoplastic cells of TME, such as tumor-associated macrophages (TAMs) and tumor-associated neutrophils (TANs), that further support cancer maintenance and progression. In addition, an attempt has been made to focus on signal transduction pathways such as the nuclear factor kappa-light-chain-enhancer of activated B cells (NF-κB), the Janus kinase/signal transducer and activator of transcription (JAK/STAT), the mitogen-activated protein kinase (MAPK), the phosphatidylinositol-3-kinase/Akt/ the mammalian target of rapamycin (PI3K/Akt/mTOR), and Toll-like Receptors (TLRs), which are activated during neuroinflammation, further supporting the role of inflammation in GBM progression and maintenance. Moreover, the present review also focuses on the potential therapeutic benefits of targeting these cytokines, TAMs, TANs, and signaling pathways in GBM.

## 2. Glioma Microenvironment

Considerable progress has been made in the past towards understanding the role of genetic and epigenetic alterations in the initiation, development, and progression of cancer. Recently, a wealth of new information revealing an integral role of the immune system and TME in cancer biology has emerged. The immune machinery of CNS plays an important role in tumor development and keeps a check on cancer cells through immunosurveillance to a certain limit. Glioma cells suppress the immune system active in the TME by downregulating costimulatory molecules, thereby evading immune elimination [10]. Immunosuppression associated with tumors is due to the interaction among glioma cells, TAMs, regulatory T cells (Tregs), and lymphocytes (peripheral and tumor-associated) [11,12]. TME is a heterogenous system and consists of cancerous cells, immune cells (e.g., lymphocytes, granulocytes, and macrophages), inflammatory cells, fibroblasts, endothelial cells, and extracellular matrix (ECM) [13,14]. These cells secrete mediators such as cytokines, chemokines, and growth factors that regulate cell−cell interaction and functioning of various cells in the TME. Furthermore, these mediators form a proinflammatory network at the tumor site, favoring tumor development, progression, invasion, and anti-tumor immune responses [10]. Within the TME, tumor cells and TAMs have also been shown to secrete cathepsins (cysteine proteases), a family of enzymes, which play an important role in cancer progression and inflammation through the regulation of signaling transduction pathways [15]. Notably, a study has demonstrated the importance of M2-derived cathepsin proteases inhibition in attenuating the tumor-promoting effects of immune-evasive TME. Moreover, inhibition of cathepsin B, L, and S by GB 111-NH_2_ resulted in changes in cellular recycling and overexpression of autophagy and lysosomal-associated marker genes [16]. Hence, targeting of cathepsins could be a promising therapeutic strategy in GBM treatment.

## 3. Molecular Mechanism of Neuroinflammation

Inflammation is a protective physiological process for the host against pathogens, tissue repair, and regeneration after injury, as well as for tissue homeostasis regulation, whereas unresolved chronic inflammation is likely to trigger carcinogenesis in 20–25% of cancer cases [17]. Neuroinflammation is one of the critical factors responsible for tumor maintenance and progression in gliomas [18].

Important cellular and molecular components like TAMs, TANs, inflammatory mediators (e.g., cytokines, chemokines, growth factors), and pattern-recognition receptors (PRRs) are the key contributing players in the immunopathogenesis of neuroinflammation [19,20]. These inflammatory molecules are either produced locally in the brain or recruited from peripheral system following disruption of the blood−brain barrier in response to the production of proinflammatory mediators [21]. During neuroinflammation, tissue homeostasis is perturbed due to tissue trauma, and immune cells such as macrophages and mast cells are activated to release cytokines, chemokines, and matrix remodeling proteins. These mediator proteins trigger activation of surrounding stromal cells (e.g., fibroblasts, vascular cells, etc.) to recruit circulating leukocytes at the site of lesion (acute inflammation) to resolve the tissue damage [22,23]. Furthermore, other cells such as dendritic cells (DCs), which are specialized antigen-presenting cells, also actively participate in the wound healing process. DCs are the connecting link between innate and adaptive immune responses. These cells are activated in response to pathogen-associated molecular patterns (PAMPs) as well as damage-associated molecular patterns (DAMPs) due to the presence of pattern recognition receptors (PRRs). DCs further process the antigens, which subsequently activates naïve T cells to spark the immune responses [24,25]. In malignancies including GBMs, the tissue repairing cascade fails to resolve the trauma, and chronic inflammation develops. The state of chronic inflammation invokes leukocytes to secrete mitogenic growth mediators in abundance, inducing a proliferation of cancer and stromal cells [22,26]. Now, we will discuss the role of proinflammatory molecules in GBM development.

## 4. Tumorigenic Role of Inflammatory Molecules in Glioma

Glioma-derived factors such as tumor necrosis factor-alpha (TNF-α), interleukin (IL)- 1α and 1β, IL-4, IL-6, IL-8, chemokines (e.g., CXCL-12), cyclooxygenase-2 (COX-2), prostaglandin (PG) E2, and platelet-derived growth factor (PDGF) are the crucial inflammatory mediators that trigger the inflammatory cycle in GBM and also promote carcinogenesis via evading growth suppression, inducing angiogenesis and metastasis, resisting apoptosis, and maintaining cancer cells stemness [20,27,28,29]. Overexpression of IL-1α and 1β was observed in glioblastoma cell lines CCF3 and U87-MG [30]. IL-1β upregulated mRNA expression of pro-inflammatory prostaglandin COX-2 in glioma cells [31]. COX-2 and its product (PG) E2 interact with four GPCRs—PGE_2_ receptors like EP1, EP2, EP3, and EP4, and thereby enhance glioma aggressiveness by maintaining glioma cell stemness and the inflammatory microenvironment [32,33]. IL-1β binds to IL-1 receptor and switches on the NF-kB pathway, leading to persistent stimulation of pro-inflammatory genes [34]. An in vitro study showed that IL-6 induces the release of VEGF, which activates the JAK/STAT3 axis, leading to an increase in cell invasion and angiogenesis [35]. In GBM cell lines U-251 and U-87, the interaction of Ras (an oncogene) with TNFα/IL-1β was found to increase hypersecretion of IL-6/IL-8 cytokines and activation of the p38 MAPK signaling pathway, leading to the creation of an inflammatory microenvironment conducive for cancer progression [36]. Immunohistochemical studies have revealed that about 65% of primary and recurrent GBM samples were positively correlated with IL-8 levels [29]. IL-8 is a potent inducer of angiogenesis, and it directs migration of endothelial cells, further stimulating the production of proteolytic enzymes-matrix metalloproteinases (MMPs) [37]. An increased expression of CXCR4 mRNA and CXCL-12 levels has been observed in human primary glioma specimens and glioma cell lines (U87, SHG-44, and CHG-5). Furthermore, chemokine (CXCL-12) was found to increase vascular endothelial growth factor (VEGF) production by CD133^+^ glioma stem-like cells via the activation of the PI3K/Akt signaling pathway, resulting in enhanced angiogenesis and metastasis [38]. Platelet derived growth factor (PDGF) has been identified to play an important role in cancer development and is often overexpressed in GBM [39,40]. Increased expression of receptor PDGFRα was reported in 33% of recurrent GBM tissues [41]. Furthermore, an aberration in PDGF signaling activates the NF-κB, PI3K-Akt, and MAPK-ERK axis, which play an important role in cell proliferation, invasion, and resistance to cell death [39,42].

### 4.1. Role of Tumor-Associated Macrophages (TAMs) in Glioma Growth and Progression

Monocytes/macrophages belong to the family of leukocytes, which originate from bone marrow and enter the blood circulation. In the state of tissue homeostasis and inflammation, blood-circulating monocytes enter the affected tissue, where they differentiate into macrophages on exposure to pro-inflammatory cytokines, chemotactic molecules, and microbial infection [43]. A tumor milieu enriched with cytokines can polarize macrophages from one phenotype to another which are classified as classically activated (M1, anti-tumor) or alternatively activated (M2, pro-tumor) macrophages. M1 type macrophages are activated by lipopolysaccharide (LPS) or PAMPs, IFN-γ, GM-CSF, whereas M2 type are activated by interleukin (IL)-4 and IL-13 [43]. In GBM, TAMs account for about 30–50% of the infiltrating immune cells which mainly include blood-circulating monocytes and tissue resident microglia [44]. Macrophages M1 and M2 have been reported to exert pro-inflammatory and immunosuppressive effects, respectively, in glioma [45]. Tissue resident macrophages (microglia) and derived macrophages have shown to share both M1 and M2 phenotypes, which can be polarized in response to different stimuli [46]. Activated M1 macrophages elicit cytotoxicity against microbes (e.g., bacteria, viruses, etc.), anti-tumor immunity, and production of pro-inflammatory cytokines (TNF-α, IL-1, IL-6, IL-12, IFN-γ, IL-23), chemokines (CXCL9, CXCL10, CXCL11, CXCL12, CXCL16, CCL2, CCL3, CCL5), reactive oxygen/nitrogen species, and COX-2 [47,48]. In contrast, activated M2 macrophages promote immunosuppression, tumor development and progression, and angiogenesis. Macrophage M2 facilitates the recruitment of T-helper (Th) 2 cells and Tregs cells. (Th) 2 cells release cytokines such as IL-4, IL-5, and IL-10, and Tregs cells cause immune-suppression, thus eventually supporting tumor growth [49,50].

Depending upon various stimuli, TAMs are further sub-classified into four types of macrophages, namely M2a, M2b, M2c, and M2d. M2a macrophages are activated by cytokines IL-4 and IL-13 and are involved in allergy and parasite killing. M2b macrophages are activated by binding of agonists to IL receptors or Toll-like receptors (TLRs) and play an important role in Th 2 cell activation and immunoregulation. M2c macrophages are activated by IL-10 and are responsible for matrix deposition, tissue remodeling, and immunoregulation. Recently, a fourth sub-type of alternative M2d macrophages were activated by IL-6 and TLR agonists via adenosine receptors. Activation of adenosine receptors induces production of anti-inflammatory cytokines (e.g., IL-10 and IL-12) and angiogenic factors (e.g., VEGF). These studies substantiate the crucial role of TAMs in tumor maintenance, progression, and metastasis [51,52].

### 4.2. Relationship between Inflammatory Mediators and TAMs

The glioma microenvironment is enriched with inflammatory mediators/chemotactic factors such as monocyte chemoattractant proteins (MCP-1, -3), glial cell-derived neurotrophic factor (GDNF), and colony-stimulating factor (CSF-1, -2). These are responsible for recruiting TAMs to the tumor site and for the polarization of TAMs from M1 to M2 phenotypes, thereby inducing tumorigenesis [52,53,54]. High levels of IL-6 in cerebrospinal fluid have been correlated with an increased population of TAMs in GBM tissue samples [55]. Monocyte chemo-attractant protein-1, secreted by different cells like endothelial, fibroblasts, epithelial, astrocytic, monocytic, and microglial cells, was identified as the first chemotactic factor [56]. Notably, a strong correlation between infiltrating microglial cells and an increased expression of MCP-1 or CCL2 in the rat glioma model was observed. There was about a ten-fold increase in the microglial cell population in GBM noted, as compared to control samples [57]. Likewise, glioma-derived MCP-3 release assisted recruitment of TAMs in human GBM cells [58].

Glial cell-derived neurotrophic factor (GDNF) has a striking similarity with the transforming growth factor-β superfamily. This growth factor was first identified in the B49 rat glioma cell line and was found to be responsible for the survival and differentiation of neuronal cells like dopaminergic neurons in the CNS [59]. GDNF contributed directly to glioma progression via interacting with its cognate receptor, GDNF receptor-α1 [60]. Furthermore, there was about five times higher GDNF level detected in human glioma mass in comparison to normal brain tissue [60]. GDNF secreted by human and rodent gliomas was found to be a strong chemotactic factor for brain resident macrophages (microglia). Another study showed that glioma cells enclosed by hollow fibers release a variety of soluble factors only. Due to the release of GDNF from the tumor cells, surrounding microglial cells become attracted and subsequently accumulate around these hollow fibers in the mouse brain [61].

Tumor-derived macrophage colony stimulating factor (M-CSF, CSF1) and granulocyte macrophage colony stimulating factor (GM-CSF, CSF2) induce microglia accumulation and activation, as well as glioma progression [62]. These factors also interact with other pro-inflammatory cytokines such as tumor-necrosis factor (TNF) and interleukin-1 (IL-1) [63]. Increased M-CSF/CSF1 expression has been correlated with angiogenesis in different tumors. GBM derived M-CSF stimulated microglial cells to secrete insulin-like growth factor-binding protein 1 (IGFBP1) to promote angiogenesis [64]. CSF2/GM-CSF triggered microglia to enhance tumor cells infiltration capacity in human glioma [62]. Another study has demonstrated an increased migration capacity of glioma cells and resistance to apoptosis due to the presence of CSF2/GM-CSF [65].

The recruitment of TAMs raises the question of whether these cellular components actively participate in glioma progression and invasion or are just bystanders. There is now convincing evidence in support of active participation of TAMs in glioma development. Threefold increase in the migration of murine glioma cells was observed under the influence of microglial cells [66]. An in vitro study in murine microglial BV2 cells showed microglial cell activation by conditioned medium from glioma cells via upregulating mRNA expression of inducible nitric oxide synthase (iNOS), interleukin (IL)-1β, IL-6, TNF-α, and COX-2. The observed microglial activation promoted tumor growth via activation of p38 mitogen-activated protein kinase (MAPK) phosphorylation and nuclear factor-kappaB (NF-κB) transcriptional activity [67]. Some microglia-derived soluble proteins, such as stress-inducible protein 1 (STI 1), epidermal growth factor (EGF), and transforming growth factor-β (TGF-β), were found to participate actively in glioblastoma progression [52]. An increased expression of a cellular prion protein, STI 1, was recorded in infiltrated TAMs and glioma tissue in vitro as well as in vivo and was well correlated with larger tumor size [68]. An overexpression of cellular adhesion protein, vascular cell adhesion molecule-1 (VCAM-1) in human GBM cells resulted from an activation of epidermal growth factor receptor (EGFR), was found to augment communication between macrophages and tumor cells, thus promoting tumor invasion [69]. A recent study has revealed an important role of aryl hydrocarbon receptor (AHR) in TAM function and cell immunity, which is activated by GBM cell-derived kynurenine. AHR promotes CCR2 expression-mediated TAM recruitment and ectonucleotidase CD39-mediated CD8^+^ T cell dysfunction by stimulating adenosine in cooperation with CD73. These observations suggest that AHR and CD39 could be considered as potential targets for reinforcing immunity against the GBM and other solid tumors [70].

TGF-β, a cytokine highly expressed in brain tumors, has dual properties—tumor suppressing or promoting. Mutation in the TGF-β signaling pathway leads to tumor-promoting effects in different types of tumors, including gliomas [71]. About a 200-fold increase in the level of TGF-β1 was noted when rat primary microglial cells were co-cultured with glioma cells, thus leading to glioma invasion. This effect was found to be mediated via TGF-β type II receptor (TβIIR) activation [72]. IL-1β and TGF-β triggered neurosphere formation and upregulation of tumor stemness genes (Bmi-1 and nestin) in glioma LN-229 cells [73]. A preclinical study has reported a significant increase in the population of TAMs as well as the angiogenic capacity of glioma cells due to high levels of TGF-β1 [74].

Tumor-bearing mice exhibited an increased expression of M1 macrophages in the initial stages of tumor development, whereas the M2 phenotype was observed in the advanced stages of cancer [75]. The transition of TAMs into M2 state releases an abundant amount of transforming growth factor TGF-β, epithelial growth factor (EGF), matrix metalloproteinase MMP-2 and MMP-9, and IL-10, thus promoting tumor angiogenesis and invasion, as well as immune-suppressed microenvironment [76]. Additionally, the levels of M2 state markers such as CD14 and CD68 were positively correlated with glioma grade [77]. IL-1β released from the macrophage M2 phenotype caused glioma cell migration. Thereafter, IL-1β phosphorylates glycolytic enzyme glycerol-3-phosphate dehydrogenase (GPD2) and activates the phosphatidylinositol-3-kinase (PI3K) pathway, thus supporting tumor cell survival and growth [78].

Immune suppression is a characteristic feature of cancer pathophysiology [11]. Activation of NF-κB signaling is required for TAM polarization and immune suppression in GBM [79]. GM-CSF, a chemotactic factor for TAMs, has been shown to enhance the immunosuppressive activity of myeloid-derived suppressor cells through the activation of interleukin-4 receptor-α (IL-4Rα) [80]. These observations strongly suggest a crucial role of TAMs in GBM development and progression and advocate their importance as a potential target in the treatment of GBM and other malignancies.

### 4.3. Role of Tumor-Associated Neutrophils in Glioma Growth and Progression

Neutrophils are another set of cellular components involved in the host defense against infection caused by pathogens and resolution of inflammation [81]. Although neutrophils are primarily involved in host defense and tissue homeostasis, in an altered state they can contribute to chronic inflammation and tumorigenesis in a variety of cancers [82]. During the early phase of inflammation/injury, neutrophils are recruited at the site of lesion under the influence of chemotactic agents such as GM-CSF, IL-6, IL-8, and CXCL1, and secrete IL-1β, TNF-α, IL-6, IL-12, MMP-9, VEGF, and arginase-1, which eventually induce chronic inflammation, promote angiogenesis, and create an immunosuppressive state [82,83]. In inflammation, neutrophils are protected from undergoing programmed cell death (apoptosis) in the presence of pro-inflammatory mediators such as LPS, G-CSF, and IL-2, thus increasing their life expectancy [48]. Tumor-associated neutrophils (TANs) are further sub-classified into N1 phenotype (pro-inflammatory and anti-tumorigenic) and N2 phenotype (pro-tumorigenic) [84]. Polarization of N1 neutrophils to N2 phenotype can be mediated by TGF-β, while N2 polarization to N1 can be done by IFN-β. N1 neutrophils exhibit diverse biological functions such as tumor cell cytotoxicity, activation of T cells, and prevention of tumorigenesis. In contrast, N2 neutrophils promote tumor development and invasion, maintenance of cancer cell stemness, angiogenesis, and immune suppression [82]. High levels of circulating vascular endothelial growth factor (VEGF), a key regulator of endothelial cell function and angiogenesis, has been shown in neutrophils (about 50%) and platelets (about 25%) [85]. The activity of the N1 type of TANs is downregulated as a tumor progresses and predominantly acquires N2 phenotype [86].

**Figure 1:**
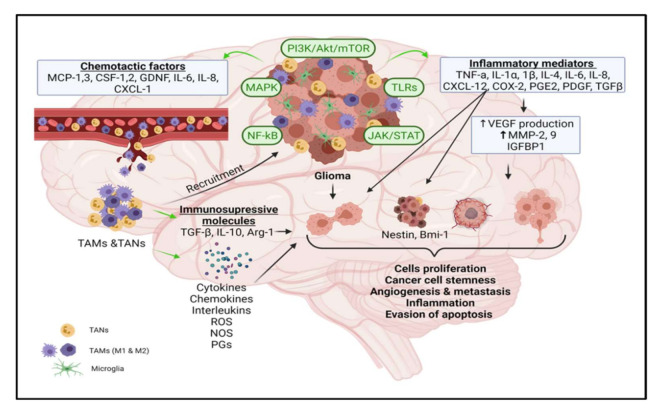
Represents tumorigenic role of inflammatory mediators, TAMs, TANs, and signaling pathways in glioma. Green color indicates activation/release. TAMs: tumor-associated macrophages; TANs: tumor-associated neutrophils; MCP-1,3: monocyte chemoattractant proteins-1,3; CSF-1,2: colony stimulating factors-1,2; GDNF: glial cell-derived neurotrophic factor; IL-1 α, β, -4, -6, -8, -10: interleukins-1 α, β, -4, -6, -8, -10; CXCL-1, -12; chemokine (C-X-C motif) ligand-1, -12; TNF- α: tumor necrosis factor- α; COX-2: cyclooxygenase-2; PGE2: prostaglandin E2; PDGF: platelet-derived growth factor; TGF β: transforming growth factor β; VEGF: vascular endothelial growth factor; MMP-2, 9: matrix metalloproteinases-2, 9; IGFBP1: insulin-like growth factor-binding protein 1; Bmi-1: B-cell specific Moloney murine virus integration site 1; Arg-1: arginase-1; NF-κB: Nuclear factor kappa-light-chain-enhancer of activated B cells; JAK/STAT: Janus kinase-signal transducer and activator of transcription; MAPK: Mitogen-activated protein kinase; PI3K/Akt/mTOR: Phosphatidylinositol-3-kinase-mammalian target of rapamycin; TLRs: Toll-like receptors.

In high-grade human GBM, the TAN infiltration rate is high compared to low grade gliomas [87]. A study on a rodent model of carcinogenesis has demonstrated a high population of neutrophils at a tumor lesion site resulting from pro-inflammatory IL-17-mediated CXCR2 axis activation [88]. Increased activity of neutrophils was correlated with high levels of interleukin IL-12 subtype (IL-12p70) in GBM patients. It was concluded that neutrophil activation is as an early indicator of tumor progression in these patients [89]. Enhanced neutrophil degranulation is associated with an increased level of arginase-1 (Arg-1) and was shown to correlate with immune suppression in GBM patients [90]. Neutrophils are phagocytosed by macrophages once infection/inflammation is resolved. Furthermore, the death of recruited neutrophils initiates the immunosuppressive cascade, similar to that involved in the tissue repair mechanism primarily mediated by type M2 macrophages [81]. As summarized in Figure 1, the above findings support the tumorigenic role of inflammatory mediators, TAMs, TANs, and associated signaling pathways in glioma.

## 5. Signal Transduction Pathways Involved in Glioma Progression

**Figure 2:**
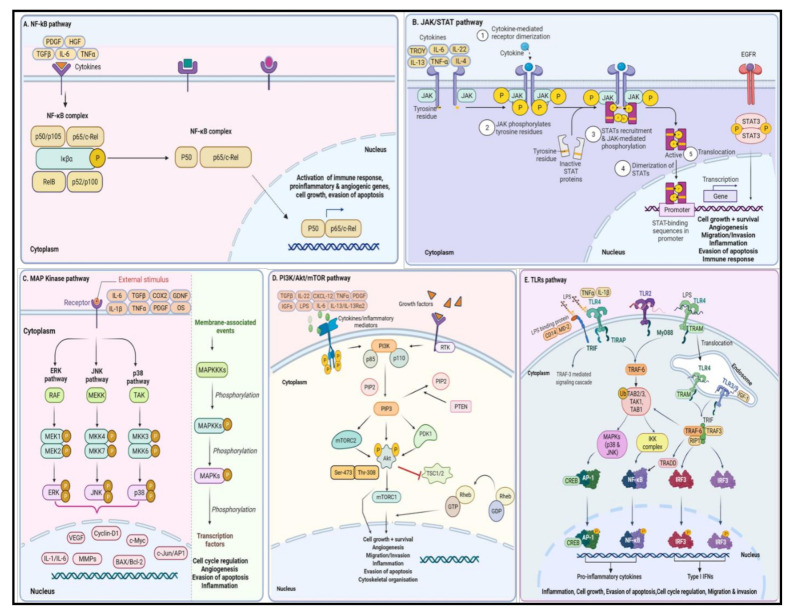
Representation of the key signaling pathways involved in the inflammation-induced glioma genesis. (**A**) NF-κB pathway; (**B**) JAK/STAT pathway; (**C**) MAP Kinase pathway; (**D**) PI3K/Akt/mTOR pathway; (**E**) TLRs pathway. NF-κB: Nuclear factor kappa-light-chain-enhancer of activated B cells; JAK/STAT: Janus kinase-signal transducer and activator of transcription; MAPK: Mitogen-activated protein kinase; PI3K/Akt/mTOR: Phosphatidylinositol-3-kinase-mammalian target of rapamycin; TLRs: Toll-like receptors; IL-1β, -4, -6, -13, -22: interleukins-1β, -4, -6, -13, -22; CXCL-12: chemokine (C-X-C motif) ligand-12; TNF- α: tumor necrosis factor- α; COX-2: cyclooxygenase-2; PDGF: platelet-derived growth factor; TGF β: transforming growth factor β; HGF: hepatocyte growth factor; OS: oxidative stress; TROY: tumor necrosis factor receptor superfamily member 19; pSTAT3: phosphorylated signal transducer and activator of transcription 3; GDNF: glial cell-derived neurotrophic factor; Iκβα: inhibitor of kappa B alpha; EGFR: epidermal growth factor receptor; ERK: extracellular signal-regulated kinase; JNK: c-Jun N-terminal kinase; VEGF: vascular endothelial growth factor; MMPs: matrix metalloproteinases; Bax/Bcl-2: Bcl-2-associated X *protein*, B-cell lymphoma 2; AP-1: activator protein-1; IGFs: insulin-like growth factors; LPS: lipopolysaccharide; PI3K: phosphoinositide 3 kinase; Akt: protein kinase B; PDK: 3-phosphoinositide-dependent kinase 1; PIP2/3: phosphatidylinositol-4, 5-bisphosphate/ phosphatidylinositol-3, 4, 5-triphosphate; mTORC1/mTORC2: mechanistic target of rapamycin complex 1 and 2; TSC1/2: tuberous sclerosis complex 1/2; PTEN: phosphatase and tensin homolog: RTK: receptor tyrosine kinase; Rheb: Ras homolog enriched in brain; CD14: cluster of differentiation 14; MD2: myeloid differentiation factor 2; MyD88: myeloid differentiation factor 88; TIRAP: TIR-containing adaptor protein; TRIF: Toll/IL-1R domain-containing adapter inducing interferon-*β*; TRAF6, -3: TNF receptor-associated factor 6, -3; TRAM: TRIF-related adaptor molecule; RIP1: receptor-interacting protein 1; TAB-1, 2/3: TAK1-binding protein-1, 2/3; TAK1: TGF-β-activated kinase; TRADD: TNF receptor type 1-associated DEATH domain protein; IGF-1: insulin growth factor-1; CREB: cAMP response element binding protein; IRF3: interferon regulatory factor 3.

The connecting link between chronic neuroinflammation and GBM progression is not fully understood. Studies undertaken in the recent past have revealed that the inflammatory microenvironment of GBM is a complex network comprised of a variety of cells, cytokines, proteins, and signaling pathways [91]. Crosstalk between glioma cells and cellular components of tumor milieu like TAMs and TANs modulates the activity of signaling pathways, which eventually promote cancer progression, tumor cell migration and invasion, and immune suppression. Some of the key pathways (Figure 2) that are activated during oncogenic events include nuclear factor kappa-light-chain-enhancer of activated B cells (NF-κB), Janus kinase-signal transducer and activator of transcription (JAK/STAT), mitogen-activated protein kinase (MAPK), phosphatidylinositol-3-kinase and the mammalian target of rapamycin (PI3K/Akt/mTOR), and Toll-like receptors (TLRs) [92,93,94,95]. Further discussion focuses on the key signaling transduction pathways that are activated during the inflammatory process and involved in GBM pathogenesis.

### 5.1. NF-κB Signaling Pathway

The nuclear factor kappa-light-chain-enhancer of activated B cell (NF-κB) pathways is comprised of key member proteins like p65 (RelA), RelB, c-Rel, p50/p105 (NF-kB1), and p52/p100 (NF-kB2). These proteins are localized in the cytoplasm of the cell and remained attenuated in the presence of inhibitor of kappa B alpha (Iκβα) protein complex [96,97]. There are two types of NF-κB signaling pathways: the classical/canonical and the non-classical/canonical. Most of the solid tumors (e.g., breast, colorectal, and glioma) exhibit high NF-κB activity. The activity of NF-κB is regulated by its signaling “classical/canonical” pathway, which is activated by inflammatory cytokines [98]. Degradation of Iκβα results in the breakdown of NF-κB complex into p50 and p65 subunits, which are translocated into the nucleus and regulate the transcription of the genes involved in immune responses, proinflammatory processes, apoptosis, angiogenesis, and metastasis. On the other hand, the non-classical/canonical NF-κB pathway selectively responds to stimuli and activates p100-sequestered NF-κB members, predominantly via translocation of p52 and RelB into the nucleus [97,99].

The role of NF-κB is important in neuroinflammation and carcinogenesis [19,100,101]. The increased activity of pro-inflammatory, angiogenic, and anti-apoptotic proteins has been linked with NF-κB activation in glioma [92]. Several studies have shown elevated NF-κB activity in different GBM models [95,102]. TGF-β has been found to upregulate miR-182 in order to prolong NF-κB activation in GBM specimens [103]. Expression of pro-angiogenic gene of IL-8 was upregulated due to NF-κB activation in glioma cells [104]. Furthermore, hepatocyte growth factor (HGF) upregulated chemokine receptor CXCR4 expression via NF-κB activation, leading to glioma cell (U87MG and LN 229) invasion [105]. The synergistic effect of cytokines TNF-α and IL-6 ascertained the belligerent nature of glioblastoma through activation of the NF-κB and STAT3 pathways [106].

### 5.2. JAK/STAT Signaling Pathway

The Janus kinase (JAK)/signal transducer and activator of transcription (STAT) pathway is one of the important pathways involved in the human pathological conditions such as inflammation, immunity, and tumorigenesis. In addition, JAK/STAT also regulates other functions such as cell proliferation, migration, and apoptosis [107,108]. The family of JAK-STAT is comprised of four members of JAK (JAK-1, -2, -3, and tyrosine kinase 2-TYK2) and seven members of STAT (STAT-1, -2, -3, -4, -5a/b, and -6) in mammals [109]. Activation of this signaling pathway requires binding of the pro-inflammatory cytokines such as IL-4, -6, -13, -22, and TNF-α to their transmembrane cytoplasmic receptors, which later undergo oligomerization process and divide into subunits like JAK2 and related mediator proteins. Furthermore, JAK2 and associated cytoplasmic receptor undergo phosphorylation, bind to tyrosine residue of STAT3 protein, and phosphorylate it. Phosphorylated STAT3 monomers undergo a transformation to form homodimers. Subsequently, these homodimers are translocated to the nucleus where they act as transcription factors to regulate the gene expression of tumor suppressor genes (e.g., p53), anti-apoptotic proteins (e.g., survivin and Bcl-2), and angiogenic factors (e.g., VEGF) [92,110,111].

In response to various stimuli such as pro-inflammatory cytokines, growth mediators and interferons, the JAK-STAT3 pathway was shown to be activated in glioma progression [112]. A study has shown an involvement of the JAK2/STAT3 axis induced by TNF-α in the neuroinflammation caused by perfluorooctanesulfonate [113]. Upregulation of orphan receptor TROY, a member of TNF receptor family, in GBM cells was correlated with high-grade tumors and poor patient survival rate. TROY increased the phosphorylation of JAK1 and STAT3 and thereby contributed to cell invasion and chemoresistance [114]. Interleukin (IL)-22, a member of pro-inflammatory cytokines, was involved in glioma genesis via JAK/STAT signaling [115]. High expression of anti-apoptotic protein, Bcl-2, observed in GBM cells was shown to be regulated by a group of interleukin members such as IL4/IL-13 through STAT3 activation [116]. In GBM cells, an interaction of nuclear EGFR with STAT3 resulted in an increase in pro-inflammatory COX-2 gene expression [117]. In addition, STAT3 was found to regulate the activity of MMP-2-mediated cell migration [118]. These observations suggest a central role of the STAT3 pathway in the pathogenesis of GBM induced by pro-inflammatory and immunosuppressive TME [107].

### 5.3. MAP Kinase Signaling Pathway

The mitogen-activated protein kinase (MAPK) pathway is another most studied signaling system involved in different types of pathological conditions such as neurodegenerative diseases and various types of malignancies, including gliomas [119]. The mammalian MAPK family consists of c-Jun NH2-terminal kinase (JNK-1, -2, -3), p38 MAPK (p38-α, -β, -γ, -δ), and extracellular signal-regulated kinase (ERK-1, -2, -4, -5) [120]. Different cellular stimuli such as peptide growth factors, oxidative stress, and cytokines are responsible for the activation of the MAPK pathway. Activation of this pathway occurs in a sequential order: initially, MAPK kinase kinase-MAPKKK phosphorylates and activates MAPKK, which in turn catalyzes the phosphorylation and activation of MAPK. Activated MAPK further phosphorylates and activates target proteins present in the nucleus-like B-cell lymphoma 2 (Bcl-2), Bcl-2-associated X *protein* (Bax), cyclin-D1, c-Jun, c-Myc, VEGF, IL-1, IL-6, and activator protein-1 (AP-1). These proteins induce tumorigenesis by promoting cell proliferation and survival, angiogenesis, inflammation, and evasion of apoptosis [121,122,123,124,125,126,127].

Glioma-derived pro-inflammatory factors, IL-1β, IL-6, TNF-α, and COX-2, were found to accelerate p38 MAPK phosphorylation [67]. TGF-β, a promoter of glioma cell migration and invasion, has been shown to increase the activity of the MAPK pathway in high-grade gliomas [128]. Activation of p38 MAPK and JNK in glioma cells stimulated the secretion of angiogenic factor VEGF, thereby inducing angiogenesis [129]. There was an induction of MMP-1 expression and GBM invasion by EGF via the MAPK pathway [130]. Another study has demonstrated glioma progression by upregulation of protein tyrosine phosphatase 1B (PTPN1) via activating the MAPK/ERK and PI3K/AKT pathways [131]. GDNF-dependent activation of ERK-1/2, JNK, and p38 MAPK has been shown to be another mechanism of glioma development [132].

### 5.4. PI3K/Akt/mTOR Signaling Pathway

The phosphatidylinositol-3-kinase (PI3K)/Akt and the mammalian target of rapamycin (mTOR) signaling axis play an important role in the regulation of cell growth and survival, angiogenesis, metastasis, invasion and neuroinflammation [133,134,135]. Receptor tyrosine kinases (RTKs) function as a second messenger system for signal transduction in the action of hormones (e.g., insulin), growth factors (e.g., EGF, TGF, and FGF), and cytokines. RTKs consist of a ligand binding domain at the cell surface, transmembrane domain, and intracellular tyrosine kinase domain. Various stimuli such as EGF and TGFα initiate the dimerization process (homo or hetero) at the cell surface, followed by phosphorylation of the intracellular tyrosine kinase domain [136]. Activated EGFR causes an activation of the downstream effectors, PI3K/Akt/mTOR [137]. PI3K, a member of lipid kinases, phosphorylates phosphatidylinositol, and it consists of a catalytic unit (p110) and a regulatory unit (p85) [138,139,140]. Upon agonist binding, phosphorylated tyrosine tail of RTKs binds to the p85 subunit of PI3K and subsequently releases the catalytic subunit p110. Furthermore, the activated p110 phosphorylates phosphatidylinositol-4, 5-bisphosphate (PIP2) to the second messenger phosphatidylinositol-3, 4, 5-triphosphate (PIP3). The tumor suppressor, PTEN (phosphatase and tensin homolog), dephosphorylates PIP3 to PIP2 and inhibits PI3K-mediated Akt activation. Therefore, loss of the function of PTEN leads to the formation of PIP3 [141,142,143]. Continuous accumulation of PIP3 leads to the recruitment and phosphorylation of downstream Akt at serine (Ser-473)/threonine (Thr-308) kinase sites. Activated Akt further mediate its response (e.g., synthesis of proteins and ribosome) through its downstream effector rapamycin-sensitive mTOR-complex (mTORC) [144].

The mammalian target of rapamycin (mTOR) protein kinase has two components, namely mechanistic targets of rapamycin complex 1 and 2 (mTORC1/mTORC2) [145,146,147]. The attachment of pro-inflammatory/immunosuppressive ligands such as IL-6, -13, and TGF-β to their cognate receptors activates Akt. Activated Akt phosphorylates and inactivates tuberous sclerosis complex (TSC1/TSC2), resulting in an initiation of mTORC1-mediated functions. Furthermore, mTORC1 interacts with its two main substrates like eukaryotic initiation factor 4E (eIF4E)-binding proteins (4E-BPs) and the ribosomal S6 kinases (S6Ks) 1 and 2, and promotes protein synthesis, cell growth, cell cycle progression, and metabolism [148,149,150]. The mTORC2 phosphorylates Akt at Ser-473 and further supports tumor growth, survival, and cytoskeletal organization [151]. An aberration in the PI3K/Akt/mTOR signaling pathway has been linked to cancer development, neuroinflammation, and dysregulation of microglia function [152].

Genomic alterations such as amplification of EGFR gene or mutation and methylation of PTEN tumor suppressor gene have been observed in 50–70% of glioblastoma cases and correlate with the deregulation of the PI3K/Akt pathway [153,154]. A total of 39% of tumor associated-microglia/macrophages overexpressed phosphorylated-mTOR in human GBM tissues [155]. In murine N9 microglial cells neuroinflammation was induced by LPS, which was found to increase the levels of AKT, mTOR, and p70S6K proteins [156]. Pro-inflammatory agonists (LPS/TNF-α) were found to upregulate mRNA expression of retinoic acid-induced 14 protein (RAI14) and associated inflammation in GBM cells via mTOR-mediated-NFκB activation [157]. Angiogenesis and cell migration are the characteristic features of cancer cells in a variety of neoplasms including GBMs. Another study has shown a positive correlation between the expression of an oncogenic pituitary tumor-transforming gene 1 (PTTG1) and that of angiogenic genes (e.g., HIF-α, VEGF, and PDGF-B), cell migrating genes (e.g., MMP2, MMP9, and MMP14), and tumor suppressing genes (e.g., p53 and p21) in glioma cells. Notably, all these oncogenic effects were regulated by the TGF-β/PI3K/Akt/mTOR pathway [158].

Invasive properties of human glioblastoma U87 cells were increased due to the up-regulation of hypoxia inducible factor-1α (HIF-1α) expression. Furthermore, a hypoxic environment caused a significant up-expression of Akt (p-Akt) and p70S6K proteins, which are the hallmarks of PI3K/Akt/mTOR pathway activation [159]. Interleukins exhibit cancer-promoting properties, cell proliferation, metastasis, and apoptosis. A study has demonstrated that the secretion of interleukin-22 by the immune cells of TME was responsible for the survival of human GBM cells and evasion of apoptosis. In addition, IL-22 treated glioma cells triggered activation of STAT3 and PI3K-Akt phosphorylation and increased anti-apoptotic protein Bcl-xL level [160]. The binding of IL-13 with its cognate receptor, interleukin-13 receptor subunit alpha-2 (IL-13Rα2), has a mechanistic role in tumor growth, invasion, and metastasis via phosphorylation and activation of PI3K, Akt, and mTOR proteins [161]. In vitro and in vivo studies have suggested that the interaction of Akt with IL-6 depends upon autocrinal/paracrinal release of IL-6. Autocrinal/paracrinal secretion of IL-6 increased Akt activity, while phosphorylated Akt enhanced IL-6 secretion to the TME, thereby resulting in the suppression of proapoptotic proteins such as cleaved PARP and caspase-3. The interaction of pro-inflammatory IL-6 with Akt contributed to chemotherapeutic resistance and glioma genesis [162]. Targeting the PI3K/Akt/mTOR axis to treat GBM and inflammatory conditions will be a topic of interest for the researchers in the future [163,164].

### 5.5. TLRs Signaling Pathway

Toll-like Receptors (TLRs) are a crucial group of pattern recognition receptors (PRRs) that can recognize PAMPs and DAMPs, generated by a wide array of molecules derived from pathogens and hosts [165,166]. Of note, TLRs play an important role in promoting neuroinflammation observed in the neurological diseases and malignancies, such as GBM [93,167]. Several studies have suggested immunotherapy—an upcoming promising therapeutic approach for treating different types of malignancies, including gliomas. In order to reinforce immunostimulatory response against the cancerous cells, the development and use of TLR agonists as adjuvant for cancer vaccines are under investigation [168,169]. However, one of the biggest challenges in stimulating TLRs is their dual role in cancer biology. For instance, during cancer development, immunosuppressed microenvironments contribute to cancer progression. TLR activators showed promising results in activating immune responses via immune cell maturation, expansion, and migration in pre-clinical and clinical studies. On the other hand, activated TLRs also possess tumor-promoting properties. These receptors are also expressed in glioma cells and immune cells of TME, and their activation creates an inflammatory milieu, favorable for tumor maintenance and progression.

#### 5.5.1. TLR Expression in the Brain

Different kinds of TLRs are expressed in the cellular entities of the brain. For example, microglial cells express a group of TLRs (TLR-1, -2, -3, -4, -5, -7, -8, -9, and co-receptor CD14); astrocytes express TLR-2, -3, -4, and -9, and oligodendrocytes express TLR-2, -3, -4. The neuronal cells also express mRNAs of TLR1-TLR9 and proteins of TLR-2, TLR-3, TLR-4, and TLR-6 [93,166]. On the other hand, in pathological conditions such as glioma genesis, the tumor cells express TLR-2, -4, and -9 [93]. These receptors require specific ligands (generally, molecules of microbial origin) for activation. Agonists for TLR-1, -2, and TLR-6 are lipopeptides; TLR-4 is primarily activated by LPS; TLR-5 is activated by flagellin; TLR-3 is activated by poly I:C, a double-stranded RNA (dsRNA) analogue; TLR-7, -8, and TLR-9 are activated by single-stranded RNA and its synthetic analogues resiquimod, imiquimod, loxoribine, and DNA containing the CpG motif [170]. It is important to understand the mechanism of the generation of pro-inflammatory molecules triggered by the activation of TLRs.

#### 5.5.2. Role of TLR Axis in Glioma Development and Associated Neuroinflammation

The role of the TLR axis has been extensively investigated and discussed in the pathophysiology of tumor development and inflammation. For example, activation of the TLR-4 signaling pathway by its agonist LPS requires first a binding of LPS to lipid binding protein (LBP) and then an interaction with CD14 (glycophosphatidylinositol-anchored membrane receptor protein) and myeloid differentiation factor 2 (MD-2) molecule [171,172,173,174]. This LPS-receptor complex forms another structure known as myddosome, which comprises adaptor myeloid differentiation factor 88 (MyD88) and Toll/IL-1R domain-containing adapter inducing interferon-*β* (TRIF) to activate interleukin-1 receptor-associated kinase-2,-4 (IRAK-2,-4), TNF receptor-associated factor 6 (TRAF6), and inhibitor of nuclear factor kappa-B kinase *ε*/TANKbinding kinase 1 (IKK*ε*/TBK1) [175,176,177,178]. Subsequently, there is an activation of associated downstream intracellular effectors such as adaptor proteins, phosphatases, kinases, and the transcription factor NF-κB [96]. Activated NF-κB further regulates the transcriptional activation of pro-inflammatory cytokines (e.g., TNF-α, IL-1α, IL-6, IL-8, etc.) and other mediators involved in neuroinflammation [166]. Specific activators are responsible for each TLR activation and recruitment of different adaptor molecules. TLR-1, TLR-2, and TLR-6 recruit MyD88 and TIR-containing adaptor protein (TIRAP), whereas TLR-5, TLR-7, TLR-9, and TLR-11 utilize MyD88 only. TLR-4 can recruit four adaptors (e.g., MyD88, TIRAP, TRIF, and TRAM) [179,180], whereas TLR-3 uses only the TRIF adaptor protein for its signal transduction [170].

#### 5.5.3. Canonical (MyD88)-Dependent TLR Signaling Pathway

The two major TLR signaling pathways, namely canonical (MyD88) and non-canonical (TRIF), have been shown to be responsible for TLR signal transduction. Macrophages and DCs, upon stimulation by TLR activators, initiate MyD88-dependent signaling via recruiting IRAK proteins. Initially, IRAK-4 is activated and recruited by adaptor MyD88, followed by subsequent activation of IRAK1 and IRAK2. Interaction of MyD88 with IRAKs forms an active complex, Myddosome (MyD88-IRAK4-IRAK2), which comprises six, four, and four active molecules of MyD88, IRAK4, and IRAK2, respectively. This complex communicates with TRAF6 (an E3 ligase required for Lys63 (K63)-linked poly-ubiquitination) to facilitate phosphorylation of kinases and activation of downstream effectors. Additionally, TRAF6, E2 ubiquitin conjugating enzymes such as Ubc13 and Uev1A, and IRAK1 undergo ubiquitination to activate TAK1, TAB1, and TAB 2/3, resulting in the activation of associated pathways like NF-*κ*B, mitogen-activated protein kinase (p38 MAPK), and c-jun N-terminal kinase (JNK) which in turn increases the activity of the effector genes (e.g., CREB and AP-1), pro-inflammatory cytokines, chemokines, and interferons (IFNs) and eventually contributes to cell survival, proliferation, differentiation, and inflammation [178].

#### 5.5.4. Non-Canonical (TRIF)-Dependent TLR Signaling Pathway

Ligation of agonists to TLR-4, -2 at the cell surface or TLR-7, -8, -9 located intracellularly is responsible for the activation of the non-canonical (TRIF)-dependent signaling pathway, involved in the release of inflammatory mediators and IFNs via activation of MAPK kinases, NF-*κ*B, and interferon regulatory factor 3 (IRF3) [166,170]. Non-canonical IκB (IKKs)-related kinases such as TBK1 and IKK-ɛ are recruited by TRAF3. Subsequently, phosphorylation and translocation of IRF3 to the nucleus initiates transcriptional modification of the targeted proteins, such as type I interferons (IFNs). During this process, the role of TRAF3 as a central regulator is crucial in activating either one of the two pathways (MyD88-dependent or TRIF-dependent). Thus, TRAF 3 degradation through ubiquitination of lysine residue K-48 activates MyD88-dependent signaling and inhibits TRIF-dependent signaling, and vice versa. TRIF interaction with TRAF6 and receptor-interacting protein (RIP1) is also responsible for TLR3-mediated activation of NF-κB [181]. Interaction of TRAF6 with N-terminal TRAF-binding domain of TRIF switches on transforming growth factor beta-activated kinase 1 (TAK1) via the mechanisms involved in the MyD88-dependent signaling pathway. TRIF interaction with RIP1 is linked with K63-polyubiquitination. Moreover, interaction of RIP1 with TNF receptor type 1-associated DEATH domain protein (TRADD) is also involved in the stimulation of NF-κB axis [178]. The presence of cytoplasmic TRADD in the GBM samples was positively correlated with stem cell maintenance and poor prognosis [182]. Overall, both Myd88 and TRIF dependent signaling pathways play a crucial role in innate and adaptive inflammatory/immune responses and GBM maintenance.

#### 5.5.5. TLR Expression and Glioma Progression

Following injury and exposure to pathogens or other toxic substances, inflammatory responses occur in the brain and other tissues as part of the repair and healing process. This innate defense response involves an interaction of PAMPs and DAMPs with TLRs of sentinel innate immune cells such as macrophages as well as mast and dendritic cells to produce pro-inflammatory mediators leading to repair and healing. However, persistent and chronic inflammation triggers the development of a spectrum of neurological diseases such as Alzheimer’s disease, encephalitis, and brain tumors [183,184]. In the adult CNS, the microglial cell population represents 10–30% of the total cells. After activation by TLR-3 ligands such as Poly I:C, dsRNA, and other TLR agonists, microglial cells synthesize and secrete IL-12, TNF-α, IL-6, IL-3, IL-10, CXCL-10, IFN-β, nerve growth factor (NGF), neurotrophin (NT)-4/5, transforming growth factor β1 (TGF-β1), glial-derived neurotrophic factor (GDNF), and fibroblast growth factor 2 (FGF2), producing chronic neuroinflammation and eventually GBM progression [185,186]. Treatment of glioma cells (e.g., A172 and LN229) with pro-inflammatory cytokine TNF-α in vitro increased the expression of TLR-4 and associated accessory molecules like MyD88, TIRAP, TRAF6, and IRF-3, which in-turn stimulated NF-κB and IFN-β, leading to the creation of an inflammatory environment conducive for cell proliferation [187]. High concentration of CD133, a biomarker expressed by the CSCs, has been correlated with the population of stem cells in different types of neoplasms and used as a biomarker of disease progression [188]. Another study has found that glioma stem cells (CD133 + GSCs) isolated from glioma cell lines and human glioma tissues, when activated by TLR-4 agonist LPS, significantly increase the expression of cytokines (e.g., MCP-1, MIP-1α, TNF-α, IL-1β, IL-6, IL-10), anti-apoptotic proteins (e.g., Bcl-2), cell cycle regulatory proteins (e.g., CDK4/6 and cyclin E), and transcription factors (e.g., NF-κB), leading to cancer progression [189]. TLR-4 activation in human glioma U251 cells stimulated the production of cytokines (IL-1β, IL-6, IL-8, and TNFα) and increased the expression of stem cell markers such as CD133 and CD34 [190]. IL- 1β secreted from glioma cells was found to be responsible for the activation of TLR-4 and upregulation of high mobility group box 1 (HMGB1) protein, which eventually results in the increase of HLA-G, a non-classical HLA class I antigen that contributes to glioma immune evasion response [191].

Upregulation of the TLR-2, -4, and Myd88 signaling pathways in human glioblastoma U87 cell line has been linked with cell proliferation and invasion [192]. Activation of TLR-2 by its’ agonist peptidoglycan (PGN) in human malignant cells (U87MG) increased phosphorylation of NF-κB and cell growth [193]. Activation of MyD88-TLR-2 signaling by glioma released factors was found to increase the expression of MMP-9 of microglia [194].

Human and murine GBM cell lines such as U87-MG, U251-MG, and C6 demonstrated high cell invasion capacity after upregulation of their TLR-9-mediated signal transduction by the receptor agonist CpG dinucleotide [195]. Other studies have proposed the prognostic and diagnostic value of TLR-9 gene expression in GBM patients [196]. In vitro glioma stem cell maintenance was attributed to TLR-9 dependent STAT3 regulation [197]. During tumor development, a variety of growth factors including insulin growth factor 1 (IGF1) have been shown to activate the TLR-9 signaling pathway and promote cancer progression by increasing the cell proliferation and inhibiting apoptosis [198]. Activation of TLR-9 resulted in the expression of inflammatory cytokines (IL-1β, IL-6, IL-8) and CXCR-4, a receptor that plays an important role in cell migration [198].

## 6. Therapeutic Applications

### 6.1. Therapeutic Potential of Targeting Glioma-Derived Inflammatory Mediators

Inflammatory mediators such as cytokines, chemokines and growth factors could serve as a promising therapeutic target by natural or synthetic agents to nullify their tumor-promoting effects in GBM. For example, blocking the activity of IL-6R by tocilizumab (humanized antibody) in glioma cell line (U87MG) prevented cell proliferation through the JAK/STAT3 pathway [199]. Inhibition of both NF-κB and STAT3 by their antagonists decreased the levels of IL-6 and IL-8 and increased the anti-invasive and cytotoxic effects in GBM [200]. Blocking the activity of platelet-derived growth factor receptor β (PDGFRβ) by the antagonist Gint4.T strongly inhibited glioma cell migration, invasion, and tumor growth in vivo [201]. Bioactive triterpenoid ursolic acid (UA) isolated from *Rosmarinus officinalis* was found to suppress IL-1β/ TNF-α and downregulate MMP-9 protein in rat glioma cells, thereby preventing cancer cell proliferation [202]. Chloroquine, an anti-malarial drug, restricted glioma cell proliferation via suppression of TGF-β activity as well as expression of plasminogen activator inhibitor-1 (PAI-1) and vascular endothelial growth factor A (VEGF-A) [203]. Immunohistochemical studies have shown a significant reduction in CXCR4 expression by antagonist PRX177561, leading to delayed GBM growth [204]. The inhibition of EP2 receptor was found to reduce the COX-2/PGE-2-mediated cAMP signaling pathway, which resulted in a marked decrease in GBM cell proliferation and invasion, induced apoptosis, and cell cycle arrest at G0–G1 phase [32]. A study has demonstrated the suppression of chemotherapy-induced cytokines and lipid mediator secretion from macrophages and resultant tumor growth by COX2/soluble epoxide hydrolase (sEH) inhibitor [205].

### 6.2. Therapeutic Potential of Targeting TAMs and TANs in Glioma

The correlation between the inflammatory mediators and the cells of TME observed in cancer maintenance and growth opens the possibility of novel therapeutic strategies targeting the non-cancerous cells (TAMs and TANs) present in the TME. Strategies to block/mitigate tumor-derived factors that help in the recruitment and polarization of non-cancerous cells and/or suppress their tumorigenic/immunosuppressive properties could be beneficial against glioma progression [206,207].

Glioma-bearing mice treated with duloxetine (a serotonin-norepinephrine reuptake inhibitor) exhibited a significant reduction in the level of chemokine CCL2/MCP-1 and prevented TAM infiltration into the tumor mass [208]. The inhibition of CC5R-mediated activity by an antagonist (maraviroc-antiretroviral drug) caused a decrease in the expression of M2 marker genes (Arg-1 and IL-10) and a significant reduction in the microglia population mediated by the downregulation of the Akt signaling pathway [209]. Treatment of GBM-bearing mice with curcumin phytosome (CCP)-caused NK cells mediated the polarization of TAMs from tumorigenic M2 phenotype to tumoricidal M1 state, which was correlated with a significant reduction of M2 markers such as p-STAT3, IL-10, and Arg-1 in TAMs as well as GBM and its stem cells [210]. Lately, a novel synergistic immunotherapy strategy based on dual targeting of IL-6 and CD40 has been proposed to potentiate anti-tumor immunity in animal GBM models. According to this study, a combination of IL-6 inhibition and CD40 activation was found to reverse immunosuppressive macrophage-mediated immunosuppression, increase the sensitivity of tumors to checkpoint blockers, and extend animal survival [211].

A marked infiltration of microglial cells and GL261 glioma cell invasion in tumor-bearing mice was observed, as evident by staining with ionized calcium-binding adaptor molecule 1 (Iba1) protein and Ki67 protein, respectively. Interestingly, glioma cell invasion and microglial cell infiltration were reduced by PLX3397, a blocker of CSF-1R signaling [212]. BLZ945, a blocker of CSF-1R, was found to downregulate the expression of the M2 phenotype of TAMs and restrict glioma growth [213]. A parallel increase in the population of microglial cells and tumor cell invasion was observed in glioma cell lines LN-18 and U-87. Moreover, the colonization of microglial cells was reduced by blocking of CSF2 signaling following CSF2/CSF2Rα antagonist administration. Furthermore, when the glioma cells deficient in CSF2 were transplanted into the mouse brain, the TAM population was diminished, and the survival rate of tumor-bearing mice improved [62]. Blocking of purinergic P2X receptor 7 (P2X7R)-dependent GM-CSF activity was shown to exert a beneficial effect against GBM [214]. In conclusion, CSF1 and CSF2 are important factors involved in TAM recruitment and glioma progression and, therefore, the inhibitors of CSF1 and CSF2 signaling could be further explored for their potential beneficial effects in GBM [212].

TANs are the major carrier of angiogenic factor VEGF, which has an important role in GBM progression. Suppression of the activity of TANs by dapsone (sulphone antibiotic) caused a decrease in the level of VEGF in GBM [215]. Dapsone inhibited pro-inflammatory IL-8-mediated neutrophilia in GBM cells and subsequently restricted glioma cell migration [216]. Recently, a novel role of neutrophils has been elucidated where it acts as a cell-based drug delivery carrier (CBDDC) for liposomes containing paclitaxel (PTX), an anti-cancer drug, in glioma-bearing mice. It has been demonstrated that under inflammatory conditions, the recruitment rate of neutrophils is high at the tumor site. The inflammatory mediators stimulated the release of liposomal PTX from the neutrophils and the delivery of PTX into the remaining invading tumor cells, eventually restricting the glioma progression [217]. This approach could be further explored to arrest GBM growth.

### 6.3. Therapeutic Potential of Targeting Signaling Pathways in Glioma

#### 6.3.1. NF-κB Pathway

Several studies undertaken in the past have reported the beneficial effects of targeting NF-κB activity in cancer and inflammatory conditions. Inhibition of the NF-κB signaling pathway by a combination of its antagonist NF-κB (p65) and temozolomide (TMZ) was found to induce apoptosis and decrease GBM cell proliferation [218]. Likewise, suppression of the NF-κB (p65) subunit as well as associated target genes (cyclin-D1, iNOS, and COX-2) by pantoprazole caused a significant decrease in human and rat glioma cell viability [219]. In glioma cells, apoptosis was induced by a bioactive compound, embelin, via suppressing phosphorylation of NF-κB (p65) and its translocation to the nucleus. In addition, embelin also activated inhibitory Iκβα, which is a negative regulator of the NF-κB signaling pathway [220]. Melatonin, a potent anti-inflammatory and antioxidant neurohormone, has been shown to protect against neuroinflammation at a dose of 10 and 100 nM in glioma cell line. The anti-inflammatory effect of melatonin was mediated through the suppression of NF-κB activity, iNOS expression, and nitric oxide (NO) production [221]. The polysaccharide fraction of *K. grandifoliola*, *C. sanguinolenta,* and *C. citratus* elicited anti-inflammatory activity against an LPS-stimulated inflammatory response in macrophages RAW 264.7 and glioma U87-MG cells [222]. Inhibition of NF-κB activity by phosphorylated mutant IκBαM downregulated the expression of VEGF and IL-8 mediators as well as tumor angiogenesis of human GBM [223]. Suppressed tumor growth and a better survival rate was observed following inhibition of NF-κB (p50), a regulator of M2 macrophage polarization. Moreover, it also decreased T cell induction, making the tumor less immunosuppressive [224].

#### 6.3.2. JAK/STAT Pathway

The important role of the JAK/STAT axis in neuroinflammation, tumorigenesis, and immune responses makes it an attractive target in achieving therapeutic goals. Treatment of neuronal and microglial cells with Asiatic acid significantly reduced methamphetamine-induced neuro-inflammation by downregulating the mRNA expression of TNF-α and IL-6 through the NF-κB (p65) and STAT3 signaling pathways [225]. Curcumin was found to abrogate the inflammatory environment in LPS-stimulated BV-2 microglial cells by upregulating the expression of suppressors of cytokine signaling (SOCS) proteins and downregulating JAK2/STAT3 activation [226]. Recently, JAK antagonist pacritinib, when used in combination with TMZ, elicited GSC-specific cytotoxicity and chemo-sensitization via regulating the JAK2/STAT3 axis in human GBM tumor initiating cells implanted in mouse brains, which eventually improved survival [227]. Inhibition of STAT3 was found to induce cytotoxic T lymphocyte activation and the resultant anti-tumor immune responses [107].

Targeting STAT3 along with agonists of intrinsic pathways of apoptosis has been suggested as a therapeutic approach for glioblastoma treatment [228]. Suppression of STAT3 activity by triterpenoid oleanolic acid decreased TAM differentiation to tumorigenic M2 phenotype and GBM cell proliferation [229]. In another study, WP1066, an inhibitor of STAT-3 activity was found to upregulate CD80 and CD86 on peripheral blood mononuclear cells and TAMs isolated from normal donors and GBM patients. In addition, it also induced the release of crucial cytokines (IL-2, IL-4, IL-12, and IL-15) needed for the activation of T cell and resultant anti-tumor immune responses [230]. Bioactive agents from plants, embelin and quercetin, have been shown to be potent inhibitors of the STAT3 axis in GBM [107]. Quercetin, a herbal flavonoid, was found to inhibit GBM cell growth and migration by attenuating IL-6-mediated JAK1 and STAT3 signaling pathways [231].

#### 6.3.3. MAP Kinase Pathway

Suppression of MAPKs and interrelated pathways has been found to be an effective and beneficial strategy for controlling cancer and inflammatory conditions. Notably, osthole, a coumarin derivative from herbs, inhibited rat glioma cell proliferation via blocking the MAPK and PI3/Akt signaling pathways [232]. Interestingly, blocking the phosphorylation process of p38 MAPK, ERK1/2, and NF-κB by hesperetin suppressed neuroinflammation in microglial cells [127]. Inhibition of the MAPKs (JNK and p38MAPK) and JAK2/STAT3 pathways also suppressed microglia-mediated neuroinflammation [233]. Recently, a novel mechanism of action of p38 MAPK dependent upon mammalian circadian clock has been proposed. According to this study, specific p38 MAPK antagonists produced more pronounced anti-invasive effects and fewer toxic effects when administered at an appropriate time of the day [234]. Downregulation of the MAPK/Akt pathway was found to be beneficial against glioma cell proliferation [235]. Solasonine, a herbal anti-inflammatory glycoalkaloid, inhibited glioma cell growth by suppressing inflammatory response through regulating p-p38 and p-JNK targets of the MAPK pathway [236]. Another study has reported that inhibition of p38 MAPK was accountable for decreased production of microglia-derived inflammatory mediators (e.g., IL-1β, IL-6, and IL-8) and GBM cells invasion [237].

#### 6.3.4. PI3K/Akt/mTOR Pathway

A variety of oncogenic functions like tumor invasion, angiogenesis, polarization of macrophages, immune suppression, and inflammation also involve PI3K/Akt/mTOR as a signaling pathway, thus making it an attractive target for cancer chemotherapy. The modulation of crucial downstream molecular targets (e.g., Akt and mTOR) of this axis plays a vital role in preventing glioma progression [238]. BKM-120 (Buparlisib), a dimorpholino pyrimidine derivative and PI3K inhibitor, produced an anti-invasive effect in GBM cells [239]. A potent allosteric Akt inhibitor MK-2206 in combination with gefitinib, an inhibitor of the EGFR tyrosine kinase, significantly induced apoptosis in glioma cells [240]. Inhibition of the PI3K/mTOR pathway was shown to be helpful in overcoming immune resistance in solid tumors and improved T cell-mediated immunotherapy [241]. A study has shown that the macrophage PI3Kγ is a regulatory switch that controls immune stimulation and suppression during inflammatory responses and tumorigenesis. This study suggested that the selective inhibition of macrophage PI3Kγ stimulated and extended NF-κB activation and suppressed CCAAT/enhancer binding protein-C/EBPβ activity, thereby restoring CD8^+^ T cell activation and cytotoxicity [242]. Previous studies have suggested an important role of C/EBP proteins in immunosuppression; however, underlying mechanisms have not been well elucidated. Therefore, the role of different members of the C/EBP family such as C/EBPβ, C/EBP δ, and C/EBP-homologous protein (CHOP) could be explored further in inflammation and GBM [243]. Treatment of human glioma cells with plumbagin, a herbal naphthoquinone, inhibited cell migration via downregulating MMP-2/-9 expression and the PI3K/Akt signaling pathway [244].

The treatment of glial cells with an immunosuppressant and mTOR inhibitor (everolimus) significantly decreased mRNA levels of inflammatory cytokines IL-1β and IL-6 and reduced total RAI14 protein expression through downregulation of mTOR activity [157]. Interestingly, IL-13Rα2-depleted-U87 glioma cells exhibited a marked decrease in the expression of p-PI3K and its downstream target gene p-Akt and p-mTOR, thus restricting tumor growth [161]. Treatment of glioma activated microglial cells with mTOR blockers promoted M2 differentiation to cytotoxic M1 phenotype, as evidenced by decreased Arg-1 and IL-10 gene expression [245]. A group of mTOR antagonists (rapamycin, temsirolimus, torin-1, and PP242) significantly suppressed GBM cell invasion by blocking TME factors like IL1β, TNFα, MMP-2, and MMP-9 proteins [246].

#### 6.3.5. TLR Pathway

As discussed earlier in this review, TLR activation has been implicated in neuroinflammation and glioma progression. However, some studies have demonstrated that hyperactivation of a single receptor or multiple receptors synergistically can exert anti-glioma effects. Interestingly, prolonged activation of TLR-9 by increasing the concentration of CpG dinucleotide was found to downregulate TLR-9 expression and induce apoptosis of glioma cells, resulting in prolonged survival of mice implanted with GBM cells [247]. Synergistic TLR-3/TLR-9 activation in microglia/macrophages suppressed glioma growth mediated by interferon β release and phagocytic tumor clearance [248]. Another study has demonstrated the potentiation of chemotherapeutic and immunostimulatory effects of cyclophosphamide when used in combination with TLR-9 agonist CpG-1826 in immunocompromised mice. Synergistic treatment with CpG-1826 and cyclophosphamide exerted antitumoral effects due to the recruitment of B cells, dendritic cells, and cytotoxic T cells, while T regulatory cells were not recruited [249]. A recent study has demonstrated that TLR-4 activation polarized immunotolerant M2 phenotype macrophages to the anti-tumor M1 state and inhibited tumor progression [250]. These observations suggest that TLR activation can exert both anti- or pro-tumor responses [251].

On the other hand, there are several studies in support of achieving therapeutic benefits against glioma by suppressing the TLRs signaling axis. An antibiotic, minocycline, significantly reduced MMP-9 production and TLR-2 expression in microglial cells treated with glioma conditioned medium [194]. A novel inhibitor of TLR-2, ortho vanillin, inhibited MMP-9, MMP-14, IL-6, and iNOS expression and decreased TLR-2-mediated pro-tumor M2 phenotype of brain resident macrophages [252]. Another study has demonstrated that the decreased levels of MTI-MMP in TLR-2 knock out (KO) mice were correlated with a decrease in glioma size and better survival rate [253]. Silencing of the TRADD adaptor protein of the non-canonical (TRIF)-dependent pathway resulted in decreased NF-κB activity, which eventually produced an anti-glioma effect [182]. A recent study has demonstrated that the silencing of TLR-4 gene in the U-87MG glioma cell line inhibited cell growth and induced apoptosis [254].

### 6.4. Therapeutic Potential of Non-Steroidal Anti-Inflammatory Drugs against Glioma Progression

**Figure 3:**
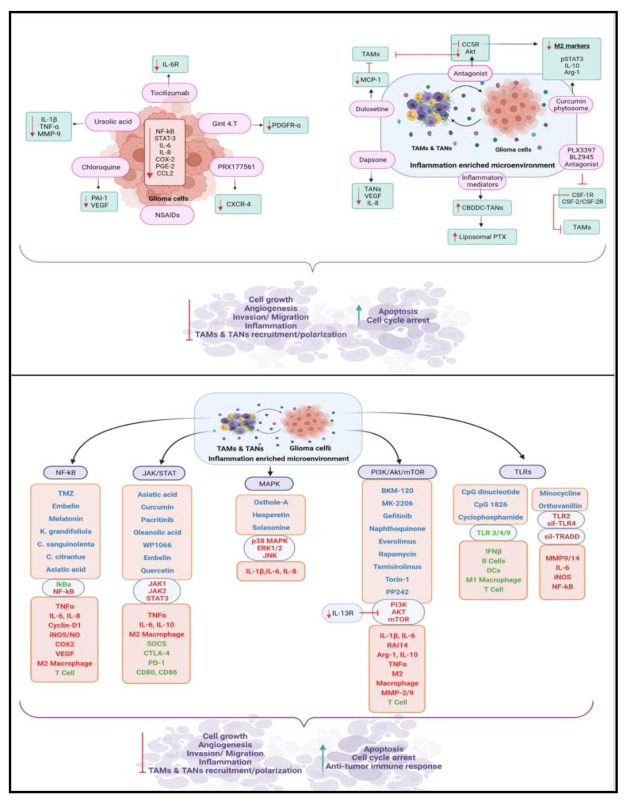
Represents the therapeutic benefits of targeting inflammatory mediators, TAMs, TANs, and signaling pathways in glioma. Red color indicates inhibition/blocking and green color shows activation/upregulation. NF-κB: Nuclear factor kappa-light-chain-enhancer of activated B cells; JAK/STAT: Janus kinase-signal transducer and activator of transcription; MAPK: Mitogen-activated protein kinase; PI3K/Akt/mTOR: Phosphatidylinositol-3-kinase-mammalian target of rapamycin; TLRs: Toll-like receptors; TAMs: tumor-associated macrophages; TANs: tumor-associated neutrophils; IL-1β, -6, -8, -10: interleukins-1β, -6, -8, -10; IL-6R: interleukin-6 receptor; TNF- α: tumor necrosis factor- α; MMP-2/9/14: matrix metalloproteinase-2/9/14; PDGFR-α: platelet-derived growth factor receptor-α; COX-2: cyclooxygenase-2; PGE2: prostaglandin E2; PAI-1: plasminogen activator inhibitor-1; VEGF: vascular endothelial growth factor; NSAIDs: non-steroidal anti-inflammatory drugs; CXCR-4: chemokine receptor type 4; STAT 3: signal transducer and activator of transcription 3; CCL2: chemokine ligand 2; MCP-1: monocyte chemoattractant protein 1; CC5R: chemokine receptor type 5; CBDDC-TANs: cell-based drug delivery carrier—tumor-associated neutrophils; PTX: paclitaxel; pSTAT3: phosphorylated signal transducer and activator of transcription 3; Arg-1: arginase-1; CSF: colony stimulating factor; Akt/PKB: protein kinase B; TMZ: temozolomide; iNOS: inducible nitric oxide synthase; NO: nitric oxide; T cell: T lymphocyte; SOCS: suppressors of cytokine signaling; CTLA-4: cytotoxic T lymphocyte associated protein-4; PD-1: programmed cell death protein-1; CD80/86: cluster of differentiation 80/86; ERK 1/2: extracellular signal-regulated kinase 1/2; JNK: c-Jun N-terminal kinase; RAI14: retinoic acid-induced 14 protein; IFN β: interferon β; B cells: B lymphocytes; DCs: dendritic cells; sil-TRADD: silencing TNF receptor type 1-associated DEATH domain protein; sil-TLR4: silencing Toll-like receptor 4.

Non-steroidal anti-inflammatory drugs (NSAIDs) such as aspirin, diclofenac, ibuprofen, and celecoxib are the most consumed drugs globally for treating debilitating inflammatory conditions such as rheumatoid arthritis and osteoarthritis [255]. Therapeutic benefits of NSAIDs have also been observed in neuroinflammation-associated disorders such as Alzheimer’s disease, Parkinson disease, and even cancer [256,257,258]. These drugs are primarily responsible for inhibiting cyclooxygenase (COX)-2 and its product, prostaglandin E2 (PGE2) [255]. Daily consumption of aspirin for ≥6 months resulted in 38% reduced risk of glioma in humans [259]. In another study, COX-2 and PGE-2 blockade inhibited glioma genesis by decreasing myeloid-derived suppressor cell (MDSC) accumulation in the TME. Treatment of tumor-bearing mice with acetylsalicylic acid (ASA) delayed glioma development and reduced MDSC-attracting chemokine CCL2 in the TME [260]. Other synthetic anti-inflammatory agents such as ibuprofen and diclofenac were found to reduce STAT3 phosphorylation in glioma cells [261,262]. Celecoxib, the selective inhibitor of COX-2, inhibited cell proliferation and induced apoptosis in human glioma cells U373 and T98G. Such treatment enhanced the activity of caspase-3 and PARP and suppressed TNF-α-induced-NF-κB (p50/p65) activation [263]. These studies suggest that the use of anti-inflammatory agents as an adjuvant in cancer chemotherapy could be useful in mitigating the inflammatory responses and immunosuppression in the TME, and thereby promoting the therapeutic effectiveness of anti-cancer drugs used for GBM. The summary of the above findings is illustrated in Figure 3.

## 7. Conclusions and Future Possibilities

Glioma TME is highly immunosuppressive and heterogeneous in nature due to inflammatory soluble mediators/factors such as cytokines, chemokines, and chemotactic factors as well as immunosuppressive cells such as TAMs, TANs, MDSCs, and Tregs. Cancer cells interact closely with the extracellular matrix and stromal cells. These immune and non-immune cells, along with the mediators/factors released from these cells, drive a chronic inflammatory and immunosuppressive tumorigenic environment. Furthermore, these cellular mediators interact with their receptors and/or TAMs/TANs and other cellular components and exert their effects via oncogenic/inflammatory signaling transduction pathways. The TME makes tumors resistant to chemo- and immune-therapy and assists in tumor maintenance and progression. Further studies towards a deeper understanding of the components of the TME and its functions are needed to develop effective and safe therapeutic strategies for the treatment of GBM and other “cold tumors”. For example, it would be worth exploring to elucidate the role of other tumor-supporting factors secreted in the glioma microenvironment, such as kynurenine, sphingosine-1-phosphate, lactate dehydrogenase, etc. [28].

Despite of a significant progress made in recent years towards an understanding of the pathophysiology of GBM and its microenvironment as well as development of potential therapeutic agents showing promise in preclinical studies and clinical trials, little success has been achieved in finding a new clinically effective drug for GBM patients. Therefore, TMZ (an alkylating agent) and bevacizumab (an VEGF monoclonal antibody), the two USA-FDA approved drugs, are still the drugs of choice for the treatment of GBM patients. Studies are underway to assess the efficacy of immune checkpoint inhibitors, tumor vaccination therapy, and chimeric antigen receptor T cell therapy. Moreover, targeted therapy focusing on the pro-inflammatory and tumorigenic mediators/factors as well as associated signaling pathways has been suggested as a promising future strategy for the treatment of GBM. Some recent studies have indicated a good scope of combination therapy in the treatment of GBM [211,264,265].

Administration of TLR agonists as immunoadjuvants in cancer therapy alone or with oncolytic viral therapy or DC-based anticancer vaccines has shown promising results in preclinical and clinical studies. However, other studies have shown limitations of employing TLR agonists as immunostimulatory agents. Improper route of administration of TLR agonists affects the pharmacokinetic and pharmacodynamic of these ligands. For example, intravenously administered agonists will be systemically distributed which can cause more side effects while decreasing the therapeutic effectiveness of the active compound [266]. In addition, the use of TLR modulators could also result in autoimmune diseases and chronic inflammation [267]. Chronic inflammation has been shown to be a critical factor for growth of malignant tumors including GBM. Moreover, TLRs agonists have been shown to produce immunosuppressive effects through the regulation of IL-10, PD-L1 expression, and Tregs, thus favoring oncogenesis [168].

Therefore, to achieve maximal therapeutic efficacy and minimal toxicity, the use of TLR agonists in the treatment of GBM or other malignancies should also take into consideration appropriate delivery systems (e.g., lipid encapsulation, peptides, etc.), dosing intervals, route of administration, and dosage forms for optimum activity and specificity of TLR agonists [266,268]. Alternatively, TLR-4 antagonists such as CXC195 and TAK-242 (resatorvid) could be explored as the anticancer agents in the treatment of GBM. These agents have been reported to possess anti-inflammatory and anti-proliferative activity and cause cell cycle arrest and apoptosis in hepatocellular carcinoma and ovarian cancer cells [269,270].
